# Intercentre reproducibility of cardiac apparent diffusion coefficient and fractional anisotropy in healthy volunteers

**DOI:** 10.1186/1532-429X-16-31

**Published:** 2014-05-06

**Authors:** Elizabeth M Tunnicliffe, Andrew D Scott, Pedro Ferreira, Rina Ariga, Laura-Ann McGill, Sonia Nielles-Vallespin, Stefan Neubauer, Dudley J Pennell, Matthew D Robson, David N Firmin

**Affiliations:** 1 Oxford Centre for Clinical Magnetic Resonance Research, Division of Cardiovascular Medicine, Radcliffe Department of Medicine, University of Oxford, Oxford, UK; 2 NIHR Cardiovascular BRU, Royal Brompton Hospital & Imperial College, London, UK; 3 National Heart Lung and Blood Institute (NHLBI), National Institutes of Health (NIH), DHHS, Bethesda, MD, USA

**Keywords:** Cardiovascular magnetic resonance, Cardiac diffusion tensor imaging, Cardiac diffusion weighted imaging

## Abstract

**Background:**

Diffusion tensor cardiac magnetic resonance (DT-CMR) enables probing of the microarchitecture of the myocardium, but the apparent diffusion coefficient (ADC) and fractional anisotropy (FA) reported in healthy volunteers have been inconsistent. The aim of this study was to validate a stimulated-echo diffusion sequence using phantoms, and to assess the intercentre reproducibility of *in-vivo* diffusion measures using the sequence.

**Methods and results:**

A stimulated-echo, cardiac-gated DT-CMR sequence with a reduced-field-of-view, single-shot EPI readout was used at two centres with 3 T MRI scanners. Four alkane phantoms with known diffusivities were scanned at a single centre using a stimulated echo sequence and a spin-echo Stejskal-Tanner diffusion sequence. The median (maximum, minimum) difference between the DT-CMR sequence and Stejskal-Tanner sequence was 0.01 (0.04, 0.0006) × 10^-3^ mm^2^/s (2%), and between the DT-CMR sequence and literature diffusivities was 0.02 (0.05, 0.006) × 10^-3^ mm^2^/s (4%).

The same ten healthy volunteers were scanned using the DT-CMR sequence at the two centres less than seven days apart. Average ADC and FA were calculated in a single mid-ventricular, short axis slice. Intercentre differences were tested for statistical significance at the p < 0.05 level using paired t-tests. The mean ADC ± standard deviation for all subjects averaged over both centres was 1.10 ± 0.06 × 10^-3^ mm^2^/s in systole and 1.20 ± 0.09 × 10^-3^ mm^2^/s in diastole; FA was 0.41 ± 0.04 in systole and 0.54 ± 0.03 in diastole. With similarly-drawn regions-of-interest, systolic ADC (difference 0.05 × 10^-3^ mm^2^/s), systolic FA (difference 0.003) and diastolic FA (difference 0.01) were not statistically significantly different between centres (p > 0.05), and only the diastolic ADC showed a statistically significant, but numerically small, difference of 0.07 × 10^-3^ mm^2^/s (p = 0.047). The intercentre, intrasubject coefficients of variance were: systolic ADC 7%, FA 6%; diastolic ADC 7%, FA 3%.

**Conclusions:**

This is the first study to demonstrate the accuracy of a stimulated-echo DT-CMR sequence in phantoms, and demonstrates the feasibility of obtaining reproducible ADC and FA in healthy volunteers at separate centres with well-matched sequences and processing.

## Background

Diffusion tensor cardiac magnetic resonance (DT-CMR) can non-invasively probe tissue microstructure through sensitivity to the small random motions of water molecules, and can provide new insights into the myoarchitecture of both healthy and diseased hearts. The apparent diffusion coefficient (ADC) and fractional anisotropy (FA) are values derived from the measured diffusion tensor and which have previously shown sensitivity to disease, including a reduced FA in patients with hypertrophic cardiomyopathy (HCM), relative to normal volunteers [1], and reduced FA with increased ADC in the area of acute and chronic myocardial infarction (MI) relative to remote myocardium in the same patients [2]. Quantitative parameters derived from the diffusion tensor, such as ADC and FA, hereafter referred to as DT-CMR invariants, are appealing as they provide the opportunity for standardised reference levels between imaging centres, enabling consistent diagnosis and staging of disease.

Standard Stejskal-Tanner diffusion imaging sequences [3] that are used clinically for neurological applications are not appropriate for cardiac applications without special post-processing, as the bulk differential motion of the heart dwarfs the diffusional motion of water molecules and leads to poor image quality. There are a number of spin-echo-based methods which have aimed to overcome this problem. These include a reduced-field-of-view, velocity compensated method [4], an acceleration and velocity compensated method with 3D SSFP readout [5], and using post-processing to remove artefacts due to motion in a large number of spin-echo diffusion images (PCATMIP) [6]. However, the most commonly-used solution to the myocardial motion problem makes use of the cyclic nature of cardiac motion, with the first and second diffusion-encoding gradients played out at the same point in two adjacent cardiac cycles when the heart has an identical position and velocity. The magnetisation is stored in the longitudinal direction in-between these gradients, and a stimulated echo is generated after the second diffusion gradient, which is sampled using an EPI readout [7,8]. This stimulated echo acquisition mode (STEAM) method is sensitive to myocardial strain over the cardiac cycle, and several methods have been proposed to address this, including strain correction [8], sweet-spot imaging [9] and using bipolar diffusion-encoding gradients [10]. However, values of ADC and FA reported for healthy volunteers using these strain-insensitive STEAM techniques have varied widely, with values between 0.6 and 0.9x10^-3^ mm^2^/s [8,10-12], and 0.33 and 0.78 [1,8,10-12] respectively. The first aim of the study was to test whether a STEAM DT-CMR sequence accurately measures ADC by validating the sequence in simple phantoms. The second aim was to determine whether the sequence, when run with the same parameters at two different centres, would yield the same ADC and FA in systole and diastole in the same ten healthy volunteers. As part of this comparison, all data were also analysed at both centres using independently developed processing.

## Methods

**Figure 1:**
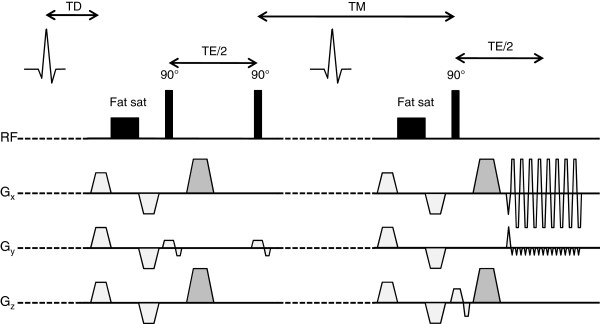
**The STEAM DT-CMR sequence used in this study.** Caption: The second fat saturation module was not included in the sequence run by centre B. The darker grey gradients control the diffusion weighting in the EPI images and were present on two of the three axes for each diffusion weighted image.

The ECG-gated STEAM DT-CMR sequence was independently implemented on two 3 tesla scanners: a Skyra (Siemens, Erlangen, Germany) at Centre B, with an anterior cardiac 18-channel array coil and a 48-channel spine array coil, of which 33 elements were used in each image; and a TIM Trio (Siemens) at Centre O, with two 16-channel cardiac array coils (anterior and posterior), of which all 32 elements were used in each image. The DT-CMR sequence used was previously described by Reese and more recently by Nielles-Vallespin et al. [8,12] and is shown in Figure 1. Briefly, it is a STEAM sequence, with monopolar diffusion encoding gradients such that diffusion encoding occurs over one complete cardiac cycle. The first two RF pulses define a reduced field-of-view in the phase encoding direction, so that the GRAPPA-accelerated single-shot EPI readout duration can be shortened, reducing the distortion in the image due to B_0_-inhomogeneity. The imaging parameters are shown in Table 1. The diffusion weighting gradients were fixed in magnitude and duration, therefore the b-values depend on the R-R interval during scanning. For a heart rate of 60 beats per minute (bpm), the diffusion weighted images had a b-value of 350 s/mm^2^. With a heart-rate of 45 bpm, the b-value increases to 467 s/mm^2^, while a heart rate of 90 bpm yields a b-value of 233 s/mm^2^. Spoilers are required in the reference image to avoid unwanted magnetization pathways and so ensure equal T1- and T2-weighting in all images. These spoilers yielded a b-value of 15 s/mm^2^ with an R-R interval of 1 s. In the diffusion-weighted images, the large diffusion-encoding gradients negate the need for spoilers. Each centre’s pulse sequence was simulated in the manufacturer’s development environment (IDEA, Siemens) and the two resulting sets of gradient waveforms were compared to ensure that they were identical.

**Table 1:** The main imaging parameters for the two sequences used in this study

**Sequence**	**STEAM DT-CMR**	**Stejskal-Tanner spin echo**
TR	2 R-R intervals (2 s for phantoms)	1 s
TE (ms)	22	66
Readout bandwidth (Hz/px)	2442	2441
Field of view (mm)	360 × 135	360 × 135
Matrix size	128 × 48	128 × 48
Voxel size (mm^3^)	2.8 × 2.8 × 8	2.8 × 2.8 × 8
b-values (s/mm^2^)	15, 350 in six directions	0, 1000 in six directions
Parallel imaging	GRAPPA, factor 2	GRAPPA, factor 2
Fat suppression	Fat saturation	Fat saturation

### Phantom validation

In order to validate the accuracy of the ADC measured by the sequence in a controlled system, alkane phantoms (Sigma Aldrich, Gillingham, UK) were imaged using the DT-CMR sequence at Centre O. The alkanes [13] were selected to provide mean diffusivities covering the range of diffusion eigenvalues previously measured in the heart [10]. These materials have no restricted diffusion characteristics, so that the measured ADC should be independent of the diffusion encoding duration. However, their viscosity is comparable to that of water *in vivo*, so that the vibrations of the patient bed due to the diffusion encoding gradients can cause rotation and shear in the fluid relative to its initial state at the start of the one-second-long diffusion encoding time, leading to artefactual signal loss in the diffusion-weighted images. It was thus necessary to place the phantoms on a separate support structure, resting on the floor and mechanically isolated from the patient bed and magnet bore. The mean diffusivity of these compounds is temperature-dependent, so the phantoms were placed in a water-bath and the bath temperature monitored during the scan using MR-compatible fibre optic temperature probes (T1 temperature probe and Reflex signal conditioning system, Neoptix, Quebec City, Canada). The scan parameters were identical for the phantom scans as for the volunteer scans, except for the slice orientation and adjustment volume. The phantoms were imaged as supplied, with a volume of 100 ml (undecane and dodecane) or mass of 100 g (tridecane, pentadecane) in glass bottles. A coronal slice through the four bottles, each stood on its base, was selected above the level of fluid in the water bath, to avoid large chemical shift artefacts (the primary spectral peak of these alkanes lies at 1.3 ppm [13]). For comparison, the diffusivity was also measured using a standard Stejskal-Tanner spin-echo diffusion sequence, again with EPI readout, the parameters of which are also included in Table 1. The phantom images were processed using the same program as the in-vivo images (see below), and mean ADC for each phantom calculated in a user-defined ROI, avoiding obvious artefacts.

### Human inter-centre comparison

For *in-vivo* comparisons, ten healthy volunteers, 7 men and 3 women aged between 23 and 58 years, underwent DT-CMR scans at the two centres, separated by a maximum of 7 days (mean 4.3 days). The study was approved by the local ethics committees and all subjects gave written, informed consent. Each scan began with standard cardiac localisers to determine the short axis orientation and a cardiac cine to determine peak systole and end diastole. Peak systole was chosen as it is likely to be of interest in studying disease: the thickened myocardium at this point in the cardiac cycle offsets, to an extent, the relatively low spatial resolution of the technique. Subject-specific second-order shimming was carried out using an adjustment volume covering the whole left ventricle. A single mid-ventricular, short axis slice was selected in each subject, 4 cm from the mitral annulus. Slice location was matched as closely as possible between the two centres and trigger times were also matched.

Each breathhold in the DT-CMR acquisition consisted of 18 heartbeats (HB):

• 2 HB: phase correction lines

• 2 HB: external GRAPPA reference lines

• 2 HB: b = 15 s/mm^2^ reference image

• 12 HB: b = 350 s/mm^2^; 6 diffusion encoding directions

This was repeated 8 times, to obtain 8 averages, in both systole and diastole. Tagging was used to track the slice location between systole and diastole so that the tissue imaged was the same, as far as possible. Line tags were prescribed perpendicular to the long axis of the ventricle in horizontal and vertical long axis views. The diastolic DT-CMR slice location relative to the tags at the diastolic trigger delay was visually matched to the systolic DT-CMR slice location relative to the same tags at the systolic trigger delay.

Both centres independently implemented offline software to analyse the resulting diffusion images in Matlab (Mathworks, Natick, MA). The timestamp on each image was used to calculate the correct b-value based on the subject’s heart rate, and each image in a set of systole or diastole was registered to the first reference image using a single-step discrete Fourier transform method [14]. For each average, consisting of one reference image and six diffusion encoding directions, six diffusion maps were calculated, one for each direction. Due to the necessary spoilers, the b-value of the reference image is non-zero (~15 s/mm^2^), and this was accounted for by subtracting the reference b-value from the b-value of the diffusion-weighted image when calculating the diffusion maps. In each subject and cardiac phase, the 48 maps (six directions and eight averages) were then used to determine the diffusion tensor at each pixel using the H-matrix-based method of Kingsley [15], from which the eigenvalues, *λ*_i_, were derived and values for the ADC and FA were calculated, defined as [16]:

$$\mathrm{ADC}=\frac{\Sigma_{i=1}^{3}\;\lambda_{i}}{3},$$

And

$$\mathrm{FA}=\sqrt{\frac{3{\sum_{i=1}^{3}\left(\lambda_{i}-\mathrm{ADC}\right)}^{2}}{2{\sum_{i=1}^{3}\lambda_{i}}^{2}}}.$$

No strain correction was carried out in the post-processing. The helix angle (HA), defined as the angle between the primary eigenvector and the tangent to the local epicardial wall in the short axis plane, was also calculated. ROIs were defined over the left ventricular myocardium, avoiding the papillary muscles, and the average ADC and FA was calculated within these regions. To obtain a single value relating to helix angle, which could be compared between centres, the approximately linear evolution of helix angle through the myocardial wall, seen both *in *[8] and *ex vivo *[17], was used. Radial lines were automatically drawn from the centre of the left ventricle to each pixel on the epicardial border, and the helix angle values within the ROI along each profile were fit to a straight line. The gradient of each linear fit, in degrees per mm, was averaged over all radial profiles to obtain the average radial gradient of the helix through the myocardial wall. The average number of interpolated image pixels used to calculate the gradient was between 5 and 7 in diastole, and 8 and 10 in systole.

Pixels with negative eigenvalues, which violate the assumption that the tensor is positive definite, were excluded from the analysis as a basic quality control mechanism. Both centres analysed all data, so that for each subject and cardiac phase, there were four possible combinations of acquisition and analysis: two acquired and analysed at the same centre, and two acquired at one centre and analysed at the other.

**Figure 2:**
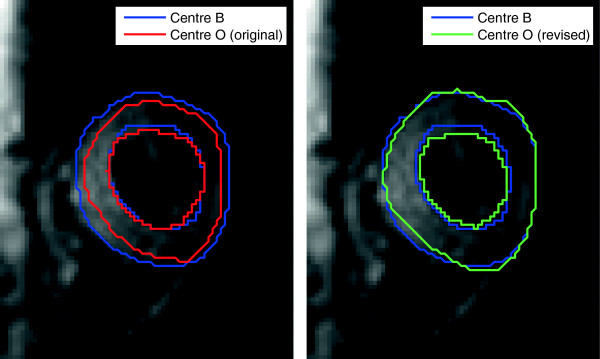
**Differences between regions of interest at the two centres.** Caption: Left shows the ROIs from the two centres as originally drawn. On the right the blue curves are the same and the green shows the better matched revised ROI from Centre O, referred to as similar ROIs in the text.

Initial analysis showed a bias between the DT-CMR invariants between the two centres. An obvious difference between the two analyses was the ROIs, examples of which are shown in Figure 2, with Centre B drawing ROIs excluding the papillary muscles and endocardial border but extending out to the full epicardium, while Centre O excluded the epicardial border. Centre O thus drew new ROIs including the full epicardium (which are referred to as “similar ROIs” in the rest of this work) and the statistics were recalculated.

### Statistics

Bland-Altman analysis [18] was carried out on the data from the two centres, including calculating limits of agreement for each metric, and paired t-tests were used to determine the statistical significance of any difference. To further investigate the differences in results between the two centres, in particular whether differences were consistently due to acquisition, the data acquired at one centre was averaged over the two analyses, and compared to the data acquired at the other centre, averaged over the two analyses. An analogous process was used to detect differences in analysis. Paired t-tests were again used to test for statistically significant differences in acquisition or analysis. The methods were deemed to be statistically significantly different when p < 0.05. Multiple comparison corrections were not used as they are not strictly appropriate when comparing multiple outcomes, and would have made the statistical tests less sensitive to any differences between the methods [19]. The mean intrasubject, intercentre coefficient of variation was calculated for each metric.

A paired t-test was used to identify any differences between the RR-intervals of the subjects when scanned in the two centres, or in the trigger delay.

## Results

### Phantoms

**Figure 3:**
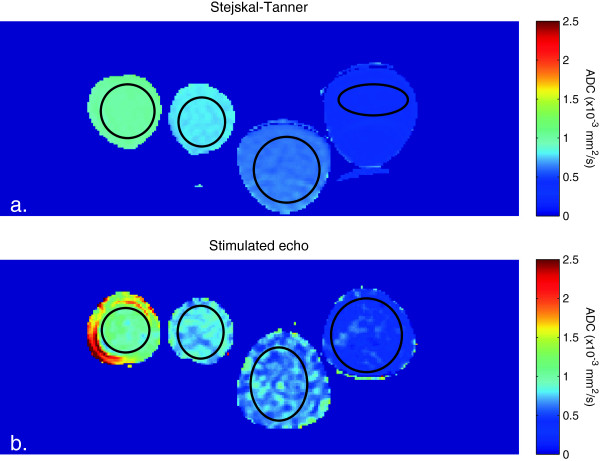
**ADC maps of the four alkane phantoms.** Caption: ADC maps acquired using **a)** the reference Stejskal-Tanner sequence and **b)** the STEAM DT-CMR sequence, showing good agreement between the two methods. The black outlines show the ROIs used to assess the ADC in each phantom.

The recorded temperature during the measurements was 19.3°C. ADC maps from the reference Stejskal-Tanner sequence and the STEAM DT-CMR sequence are shown in Figure 3, including the ROIs used for analysis. The STEAM method has lower SNR leading to some inhomogeneity in the ADC map, as well as some artefacts, particularly on the edges of the phantoms. These arise from incomplete suppression of residual rotational and shear flow, despite the mechanical isolation of the phantoms from the table vibrations. The numerical results are shown in Table 2, along with the relaxation times of the phantoms and their expected diffusivities at 19.3°C, calculated from the quadratically-corrected Arrhenius expression of Tofts et al. [13]. The Stejskal-Tanner sequence gives diffusivities within 0.02 × 10^-3^ mm^2^/s (2.5%) of the reference values for all four phantoms. There is excellent agreement between the reference values, Stejskal-Tanner and STEAM sequence results for the two highest-diffusivity alkanes, with reasonable agreement for the less diffusive phantoms, with a maximum difference between reference and mean measured diffusivity using the STEAM DT-CMR sequence of 0.05 × 10^-3^ mm^2^/s (7.5%).

**Table 2:** Characteristics of and results for the alkanes used to validate ADC values

			**Mean diffusivity (×10**^**-3**^ **mm**^**2**^**/s)**		
	**T1 (ms)**	**T2 (ms)**	**Literature value at 19.3°C **[13]	**Stejskal-Tanner**	**STEAM DT-CMR**
Undecane	1331	204	0.994	1.00 ± 0.02	1.00 ± 0.10
Dodecane	1160	163	0.770	0.79 ± 0.01	0.80 ± 0.07
Tridecane	999	173	0.623	0.63 ± 0.02	0.67 ± 0.09
Pentadecane	751	178	0.395	0.40 ± 0.01	0.42 ± 0.10

Caption: MR parameters and expected diffusivities of the alkanes used [13] and the measured diffusivities of the four alkane phantoms, presented as mean ± standard deviation over ROI, showing good agreement between all three.

### Healthy volunteers

**Figure 4:**
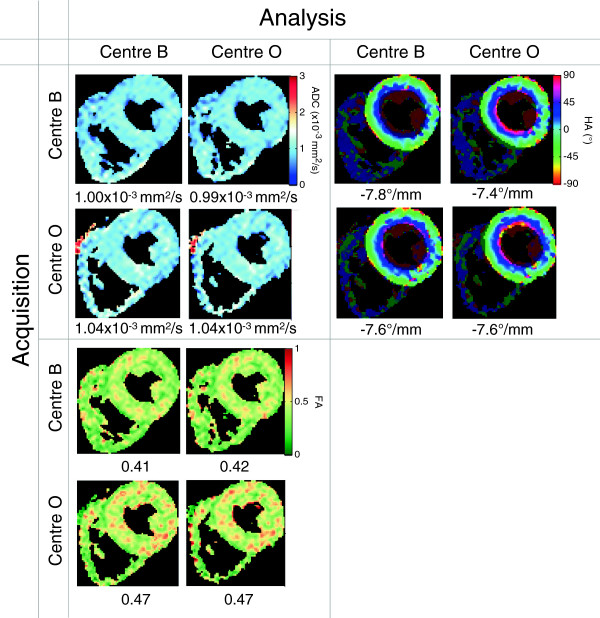
**Sample results in systole from one of the healthy volunteers in this study.** Caption: This demonstrates visually the similarity between both acquisition and analysis at the two centres. The ROIs used are shown by the brighter areas on the helix angle maps and the value below each map is the average over the ROI.

An example set of images from a typical volunteer is shown in Figure 4. The DT-CMR invariants are shown for each centre acquiring and analysing its own data in Table 3. All DT-CMR invariants for each subject, acquired and analysed at each centre, are included in Additional file 1. The mean ± standard deviation trigger delay in systole was 336 ± 53 ms and in diastole was 790 ± 100 ms, and the mean RR-interval was 1090 ± 50 ms. There was no significant difference between centres in RR-interval (mean difference 10 ms, p = 0.68) or trigger delay (mean difference 0 ms, p = 1). The mean percentage of pixels with negative eigenvalues in the myocardial ROI was 0.3%.

**Table 3:** Diffusion values measured in this study

	**Systolic**			**Diastolic**		
	**ADC**	**FA**	**HA grad**	**ADC**	**FA**	**HA grad**
	**(×10**^**-3**^ **mm**^**2**^**/s)**		**(°/mm)**	**(×10**^**-3**^ **mm**^**2**^**/s)**		**(°/mm)**
Centre B	1.06 ± 0.06	0.41 ± 0.05	-8.7 ± 1.4	1.17 ± 0.14	0.54 ± 0.04	-10.1 ± 0.9
Centre O	1.13 ± 0.13	0.40 ± 0.07	-9.0 ± 1.4	1.26 ± 0.16	0.55 ± 0.03	-10.2 ± 0.8

Caption: The median value and interquartile range for the DT-CMR invariants at each centre, along with the helix angle gradient across the myocardium, as measured in the same 10 healthy volunteers.

**Figure 5:**
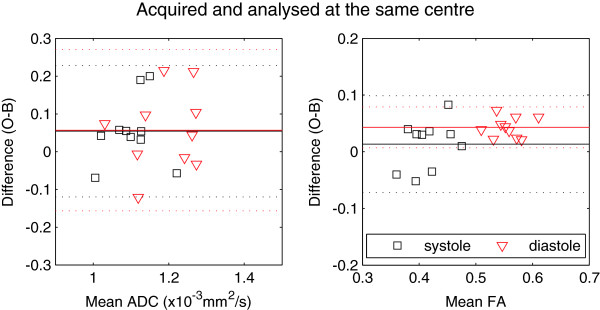
**Bland-Altman plots to compare the diffusion metrics obtained by each centre independently.** Caption: Based on a paired t-test, the diastolic FA is highly statistically significantly different (p < 0.001, difference Δ = 0.04), while systolic ADC (Δ = 0.05 × 10^-3^ mm^2^/s), FA (Δ = 0.01) and diastolic ADC (Δ = 0.06 × 10^-3^ mm^2^/s) are not found to be statistically different.

Bland-Altman plots showing the main comparison of ADC and FA between centres (acquisition and analysis at the same centre), based on the initial ROIs, are shown in Figure 5. In both systole and diastole, ADC and FA are higher at centre O than centre B, but the difference only reaches statistical significance for diastolic FA (mean bias 0.04, p < 0.001).

**Figure 6:**
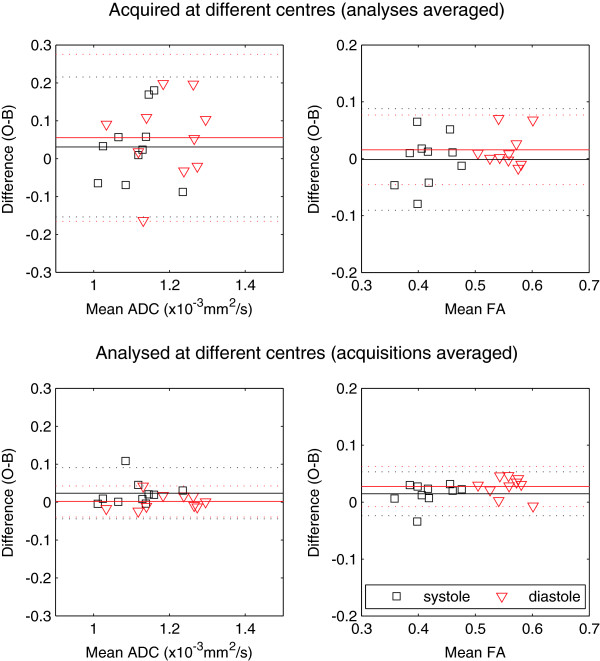
**Bland-Altman plots to illustrate the differences arising from the acquisition and analysis in the two centres.** Caption: The upper plots show the difference between the acquisitions at the two centres, averaged over the two analyses, and vice versa for the lower plots. The differences in the analysis of FA motivated the use of more similar ROIs, along with the t-test results, which showed a significant effect of analysis on the diastolic FA (p = <0.001).

Further Bland-Altman plots are included in Figure 6, showing the difference in acquisition and analysis separately. This showed that the bias in the results primarily arose from the analysis, particularly for diastolic FA, which was borne out by the results from the t-tests, which showed that the difference in analysis between the two centres was significant (difference 0.03, p < 0.001). As mentioned in the methods section above, the ROIs were re-defined at Centre O to match more closely the approach taken by Centre B, (hereafter referred to as “similar ROIs”) with the aim of reducing this difference in analysis.

**Figure 7:**
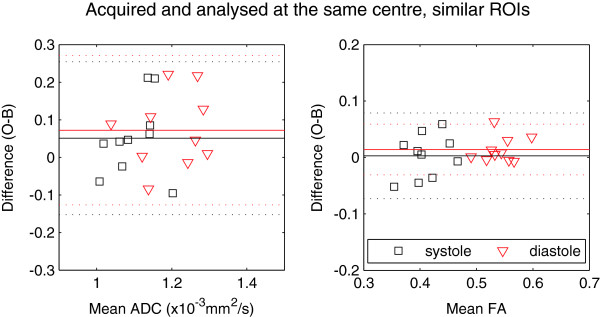
**The same plots as in Figure** 5**, but analysed using similar ROIs at the two centres.** Caption: Differences are not statistically significant between systolic ADC (Δ = 0.05 × 10^-3^ mm^2^/s), FA (Δ = 0.003) or diastolic FA(Δ = 0.01). There is a marginally statistically significant difference between diastolic ADC measurements (Δ = 0.07 × 10^-3^ mm^2^/s, p = 0.047).

**Figure 8:**
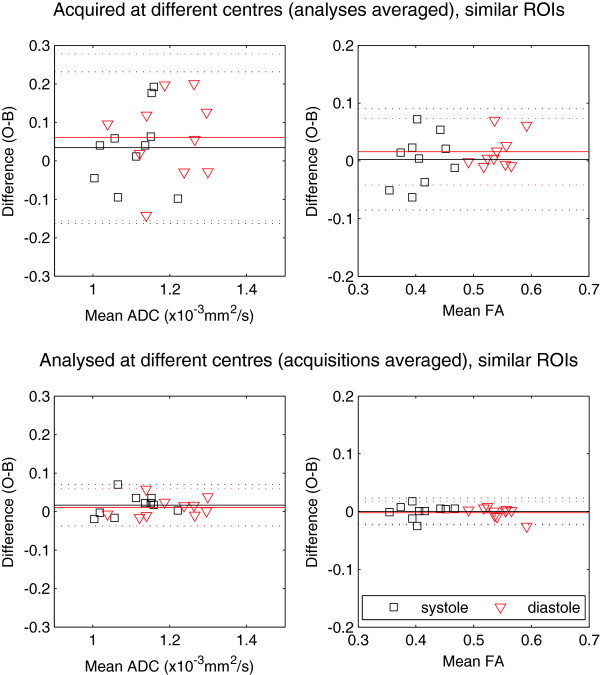
**The analogue of Figure** 6**, with the similar ROIs.** Caption: These plots show the reduced differences in analysis, which are now small compared to those from acquisition.

Bland-Altman plots using the similar ROIs are shown in Figures 7 and 8 (equivalent to Figures 5 and 6). In Figure 7, where we compare FA and ADC acquired and analysed at the same centre using similar ROIs, the bias in diastolic FA is reduced to 0.01, which is reflected in the paired t-test results: neither the systolic (difference 0.003) nor diastolic FA differences were statistically significant. Systolic and diastolic ADC showed similar differences between the two centres, at 0.05 and 0.07 × 10^-3^ mm^2^/s, with only the diastolic ADC difference being marginally statistically significant (p = 0.047). None of the acquisition- or analysis-specific t-tests showed statistically significant differences for any parameter when similar ROIs were used. Figure 8 shows that the mean differences due to analysis are small when similar ROIs are used (<0.02 × 10^-3^ mm^2^/s for ADC and <0.002 for FA) and all the points are tightly clustered around the mean. The remaining differences arise from acquisition: 0.03 and 0.06 × 10^-3^ mm^2^/s for ADC in systole and diastole, and 0.003 and 0.02 for FA in systole and diastole.

The helix angle gradient, calculated using the similar ROIs, showed agreement between the two centres, with a mean of -9.1 ± 1.0°/mm and difference of -0.4°/mm (p = 0.33) in systole, and a mean of -10.3 ± 0.8°/mm and difference of 0.2°/mm (p = 0.53) in diastole. The CoV of the HA gradient is 11% in systole and 8% in diastole.

Table 4 shows limits of agreement calculated for ADC and FA in systole and diastole using similar ROIs with acquisition and analysis in the same centre.

**Table 4:** Limits of agreement for the main parameters in this study

		**ADC**	**ADC CoV**	**FA**	**FA CoV**
		**(x10**^**-3**^ **mm**^**2**^**/s)**			
Previous work	Systole	0.7 to 1.0		0.53 to 0.65	
This work	Systole	1.0 to 1.4	7%	0.34 to 0.49	6%
	Diastole	1.1 to 1.5	7%	0.51 to 0.61	3%

Caption: Limits of agreement are defined as mean ± 1.96(standard deviation of the differences), and are compared to previously reported values [12], along with the intrasubject, intercentre mean coefficient of variance (CoV) measured in this study.

## Discussion

### Phantoms

These are the first published phantom validation experiments for a STEAM-based diffusion sequence designed for DT-CMR. We have used alkane phantoms and have found that the sequence generates values for diffusivity which are all within 7.5% of the correct values. The DT-CMR sequence agreed with both references (values from the literature, and from a Stejskal-Tanner diffusion sequence) to within 0.05 × 10^-3^ mm^2^/s for all four phantoms. The cardiac DT-CMR sequence, when it differs from the reference and Stejskal-Tanner values, overestimates the diffusivity. Any net rotation or shear due to fluid flow over the 1 s diffusion encoding time in the STEAM sequence will tend to lead to an overestimation of the diffusivity. While the phantoms were left to settle for one hour before scanning, even with the mechanically isolating support structure it was not possible to completely remove all gradient-induced vibration from the phantoms.

An obvious solution to the problem of gradient induced motion would be to use a material including a gelling agent, for example sucrose-doped agarose phantoms [20]. These would enable a range of physiological diffusivities to be generated without any artefacts in the diffusion acquisition due to bulk flow. However, agarose phantoms display some restricted diffusion characteristics [21], meaning that the diffusivity cannot be uniquely defined. In particular, measurements with different diffusion encoding times will yield different ADCs, thus making a direct comparison between a reference Stejskal-Tanner measurement and one using the STEAM sequence impossible.

### Healthy volunteers

The results reported here show that the ADC and FA values between the two centres are consistent, when similar ROIs are used. The initial, highly statistically significant difference in diastolic FA between the two centres could be ascribed to the exclusion of the epicardial border from the ROIs drawn at centre O. When similar ROIs are used, there is a statistically significant difference between diastolic ADC of 6%, but the statistical significance is marginal (p = 0.047) even in this n = 10 paired study. In addition, the limits of agreement (shown in Table 4) are comparable in size to those previously reported in a single-centre reproducibility study on healthy volunteers [12].

The helix angle gradient, measured in degrees per mm, showed no statistically significant difference between the two centres. There is no consistent approach to global helix angle metrics in the literature thus far. Both the proportion of fibres with helix angles in a certain range [1] and the average helix angle in the epi-, meso- and endocardium [22] are liable to be extremely sensitive to ROI definition so were not investigated in this study. The results reported here suggest that the helix angle gradient may be a useful and reproducible metric. However, it should be noted that the helix angle, unlike the ADC and FA, is not a rotationally invariant property of the diffusion tensor. As such, any HA quantification depends on the accurate definition of the long axis vector and thus may be dependent on the particular slice orientation and definition of the centre of the left ventricle.

This comparison finds that the mean ADC measured at both centres in this study is higher than previously reported. A range of values from studies using comparable techniques in healthy subjects, along with the changes due to two diseases is shown in Table 5. The table also includes FA values; while these are expected to depend on voxel size and thus more variation would be expected across the literature, the differences between previous studies, as well as with this work, cannot be explained on this basis alone. There are a number of possible reasons for these discrepancies, including technical errors in the sequence or processing, strain and SNR, each of which is discussed below.

**Table 5:** Comparison of previously published ADC and FA values with those measured in this study

**Reference**	**Field strength (T)**	**Voxel size (mm**^**3**^**)**	**ADC (x10**^**-3**^ **mm**^**2**^**/s)**		**FA**		**Notes**
			**Healthy volunteers**	**Disease**	**Healthy volunteers**	**Disease**	
				**(% change)**		**(% change)**	
Reese et al. [8]	1.5	3 × 3 × 9	0.87 ± 0.11		0.65 ± 0.03		
Dou et al. [10]	1.5	4 × 4 × 12	0.60 ± 0.11		0.7 ± 0.1		
Tseng et al. [1] (HCM)	1.5	3 × 3 × 3	not reported		0.78	0.75 (-4%)	Free wall
					0.72	0.56 (-22%)	Septum
Wu et al. [2] (MI)	1.5	1.9 × 1.9 × 8	0.65 ± 0.03	0.92 (+46%)	0.33 ± 0.02	0.25 (-26%)	Acute
				0.74 (+17%)		0.27 (-21%)	Chronic
Nielles-Vallespin et al. [12]	3.0	2.9 × 2.9 × 8	0.8 ± 0.1	-	0.60 ± 0.04	-	
McGill et al. [22] (HCM)	3.0	2.9 × 2.9 × 8	-	0.73 (-9%)	-	0.62 (+3%)	
This work	3.0	2.9 × 2.9 × 8	1.10 ± 0.06		0.41 ± 0.04		Systole
			1.20 ± 0.09		0.54 ± 0.03		Diastole

Caption: This table includes mean healthy values for systolic ADC and FA (±standard deviation where stated) using monopolar STEAM DT-CMR sequences previously reported in the literature, and changes reported in two diseases: hypertrophic cardiomyopathy (HCM) and acute and chronic myocardial infarction (MI).

In this work, the possibility of a technical error in the sequence or processing has been limited by using simple alkane phantoms with a range of ADC values to validate the sequence and processing. While there was not perfect agreement between the DT-CMR sequence and the reference values, the median error over all four phantoms was less than 0.03 × 10^-3^ mm^2^/s. This is much smaller than the differences between the ADCs measured in this study and those in the literature. Simulated gradient waveforms from both sequences were compared between the two centres and found to lead to identical diffusion weighting in the sequence.

Most previous work has not discussed the diffusion weighting due to the spoilers in the reference image. If this was not taken into account in prior analyses, it is possible that this could account for some or all of the difference between previously reported data and that in this study. In particular, this is the source of the difference between this work and refs [12] and [22]. Those studies used larger spoilers than were employed here, with b = 135 s/mm^2^[23], the diffusion-weighting of which was not included in the original analysis. This led to the decreased ADC and increased FA relative to the values presented here. When these spoilers are accounted for, the data show good agreement with this work [23].

SNR is known to have a large impact on the quantification of DT-CMR data, and one of the key problems is related to the noise bias of the measured signal due to the magnitude reconstruction of the diffusion-weighted images [24,25]. This signal bias due to noise rectification depends on the number of channels in the coil, becoming larger with higher numbers of elements, as well as the image reconstruction algorithm. The current study was carried out using receive arrays with far larger numbers of elements than were available ten years ago when the original work on DT-CMR was published. Centre O used a 32 channel array, and Centre B 33 channels of two butterfly-loop arrays. This difference with much of the existing literature would be expected to bias the ADCs measured in this study downwards relative to those previously reported. Instead, here we see an increase in ADC compared to prior values, implying that coil differences between this study and previous work cannot explain the differences seen.

Strain is also known to affect the measurement of DT-CMR invariants [8] . Given that diffusion encoding always occurs over the whole cardiac cycle in a STEAM sequence, differences in the resulting DT-CMR invariants between systole and diastole when strain is not corrected for can only be attributed to the different average strain over the cardiac cycle. Over the entire cardiac cycle, strain has been shown to alter the ADC measured using a monopolar STEAM sequence by up to 35%, from ~0.53 × 10^-3^ mm^2^/s at peak systole to 0.73 × 10^-3^ mm^2^/s in very late diastole [9]. The median peak systolic and late diastolic ADCs measured in this study are 1.1 × 10^-3^ mm^2^/s and 1.2 × 10^-3^ mm^2^/s and the ADC, when not corrected for strain, is thought to oscillate over the cardiac cycle around the true value [9]. Thus we attribute the 10% difference in ADC and 30% difference in FA measured in systole and diastole seen in this work to strain effects. However, given that the median ADC values measured here in both systole and diastole exceed the maximum reported strain-corrected ADC in the literature of 0.9 × 10^-3^ mm^2^/s [8], the difference between literature values and the values in this study cannot be attributed to strain effects alone.

### Multi-centre DT-CMR and disease

Included in Table 5 are some previously reported changes in DT-CMR invariants measured in two diseases. These changes are also not always consistent between studies, but decreases in FA of around 20-25% have been observed in HCM [1] and MI [2] patients, and increases of nearly 50% in ADC in acute MI patients [2]. A semi-quantitative comparison of these changes with the intrasubject coefficients of variance shows that changes of these magnitudes would be detectable were a subject scanned at one centre and then at another: the ADC CoV is 7% and the maximum FA CoV is 6%. However, more recent work finds that the difference in FA seen in HCM may be much smaller [22], at around 3%. In that single-centre, patient study the intrasubject CoV for ten subjects over two days was 7.2% for systolic FA, more than twice as large as the change due to disease.

The comparable range encompassed by the limits of agreement seen in a single centre study [12] and here (Table 4) demonstrate that the loss in statistical power in moving from a single to a multicentre study would be small, and could easily be offset by the additional recruitment such a change would engender. In the case that the FA change in HCM is as small as 3%, the increased numbers available in a multicentre study may be required to enable the detection of differences in FA associated with, for example, ECG markers of conduction abnormalities. However, we would emphasise that these sequences were matched as closely as possible and implemented on the hardware from the same manufacturers and that deviations in acquisition or analysis can lead to larger variance between centres.

## Conclusions

In this study, we have demonstrated that a DT-CMR sequence yields correct diffusivity values in alkane phantoms. The sequence was used to obtain consistent DT-CMR data between two centres, particularly in systole, with limits of agreement comparable in size to those obtained in a single-centre reproducibility study. The ADC measured in this study in healthy volunteers in systole and diastole was larger than that found in previous published DT-CMR studies by between 20 and 60%. The definition of regions of interest in the myocardium can have a significant effect on the analysed results, particularly for the FA, so it is important to have a consistent methodology if results are to be compared.

## Competing interests

DJP is a consultant to Siemens and a shareholder and Director of Cardiovascular Imaging solutions.

## Authors’ contributions

All authors participated in the study design and read and approved the final manuscript. EMT, ADS, PF, RA, LAM, SNV, MDR and DNF contributed to image acquisition and processing. EMT carried out the statistical analysis and drafted the manuscript.

## Authors’ information

Elizabeth M Tunnicliffe and Andrew D Scott are joint first authors.

## Supplementary Material

Additional file 1 ADC and FA for each subject in the study, with acquisition and analysis at each centre. Click here for file
